# Daily rhythms of behavioral and hormonal patterns in male dromedary camels housed in boxes

**DOI:** 10.7717/peerj.3074

**Published:** 2017-03-29

**Authors:** Lydiane Aubè, Meriem Fatnassi, Davide Monaco, Touhami Khorchani, Giovanni Michele Lacalandra, Mohamed Hammadi, Barbara Padalino

**Affiliations:** 1 Livestock and Wildlife Laboratory, Arid Lands Institute (I.R.A.), Médenine, Tunisia; 2 Department of Emergency and Organ Transplantation (D.E.T.O.), Veterinary Clinics and Animal Production Section, University of Bari Aldo Moro, University of Bari, Italy; 3 Department of Veterinary Medicine, University of Bari, Valenzano (Bari), Italy

**Keywords:** Daily rhythm, Cortisol, Testosterone, Behavior, Camel

## Abstract

**Background:**

Daily rhythmicity has been observed for a number of hormonal and behavioral variables in mammals. It can be entrained by several external factors, such as light-dark cycle and scheduled feeding. In dromedary camels, daily rhythmicity has been documented only for melatonin secretion and body temperature. In this study, the daily rhythmicity of behavioral repertoire, cortisol and testosterone levels was investigated in captive male camels.

**Methods:**

Six clinically healthy male dromedary camels (*Camelus dromedarius*) were used. The animals were housed in single boxes for 24 h daily and fed twice a day. Over a period of 48 h, behavioral observations were made and blood samples taken every two hours. The data were analyzed using diurnality index, conisor analysis and PROC mixed procedure.

**Results:**

The diurnality index for rumination and lying down was close to 0 (respectively, 0.09 and 0.19), while the indices for stereotypy, standing, feeding and walking were close to 1 (respectively, 0.74, 0.84, 0.92 and 0.85). Cosinor analysis revealed daily rhythmicity for all behaviors and for cortisol levels (acrophase at 12:57) but not for testosterone. Rumination and lying down (inactive behaviors) reached a peak during the scotophase, whereas feeding, walking and stereotypy (active behaviors) reached a peak during the photophase around midday. Cortisol level and expression of stereotypies peaked before and after food distribution and were negatively correlated (*r* =  − 0.287, *P* = 0.005). Testosterone levels and expression of sexual behaviors were stimulated by the visual and olfactory contacts with the females and were positively correlated (*r* = 0.164, *P* = 0.040). Testosterone was also negatively correlated with cortisol (*r* =  − 0.297; *P* = 0.003).

**Discussion:**

These preliminary results provided new knowledge about the daily rhythm of behaviors in camels housed in boxes, suggesting that camels exhibit diurnal behavior pattern in the maintenance conditions outlined in the study. Daily rhythmicity seemed to be entrained not only by the light-dark cycle but also by scheduled feeding. The rise in stereotypy after food distribution could be due to the persistence of feeding motivation and frustration after the ingestion of food. Therefore, feeding practices should be improved to satisfy the foraging and feeding motivation of these camels. Behavioral and hormonal daily patterns in camels should be taken in consideration to adapt the management system, giving the animals more freedom during the light period and a diet richer in fiber, so as to improve reproductive performance, health and welfare.

## Introduction

A daily rhythm corresponds to a regular oscillation of behavioral and physiological variables over a period of 24 h (Refinetti, Cornélissen & Halberg, 2007). In mammals, the daily clock, located in the suprachiasmatic nuclei (SCN), regulates the metabolism and coordinates the different physiological functions, ensuring the organism is synchronized with its environment (Froy, 2011; Vitaterna, Takahashi & Turek, 2001). Daily rhythmicity is entrained or influenced by several external factors, known as ‘Zeitgeber’ (i.e., timing-cue), such as ambient temperature and light-dark cycle (Berger, 2011; Piccione & Caola, 2002). The light-dark cycle is known to be the dominant Zeitgeber entraining the daily rhythmicity of physiological and behavioral processes (Dibner, Schibler & Albrecht, 2010; Piccione & Caola, 2002). Behavioral and physiological rhythms could also be entrained, synchronized or influenced by scheduled feeding time (Boulos & Terman, 1980). Thus, limiting food access to precise times in the day could act as a Zeitgeber on behavioral and physiological rhythmicity (Castillo et al., 2004; Schibler, Ripperger & Brown, 2003).

Daily rhythmicity of body temperature, cortisol and locomotor activity has been widely studied and described in many domestic animals, using different approaches, including diurnal index, conisor analysis and analysis of variance (Becker et al., 1985; Bohák et al., 2013; De Jong et al., 2000; Fulkerson, Sawyer & Gow, 1980; Giannetto et al., 2012; Martin et al., 2010; Piccione et al., 2010; Piccione et al., 2011; Piccione, Caola & Refinetti, 2002; Piccione et al., 2008b; Refinetti & Piccione, 2005; Thun et al., 1981). Disruption of daily rhythmicity can be associated with health problems, reduced life expectancy and altered well-being of the organism (Froy, 2011; Vitaterna, Takahashi & Turek, 2001). Thus, the study of behavioral and physiological daily rhythmicity in animals provides knowledge of their daily organization and the findings are useful to ensure animals can be managed in such a way as to meet their requirement of daily rhythmicity, thus safeguarding their welfare (Berger, 2011). Good housing, good feeding, good behavior and good health are indeed the four welfare principles used to assess animal welfare (Dalla Costa et al., 2014).

Daily rhythmicity has been poorly investigated in camels. Female camels presented daily rhythmicity of body temperature which appeared to be entrained by both light-dark cycle and ambient temperature (El Allali et al., 2013). Melatonin secretion also showed a daily rhythmicity, with high levels during the dark period and low levels in the light period (Mohammed et al., 2010; Vyas et al., 1997). The number of camels reared intensively has increased rapidly as they are now more frequently used for milk and meat production worldwide (Faye & Bonnet, 2012). However, it has been reported that housed camels may develop stereotypies (Padalino et al., 2014), which are repetitive, unvarying and apparently functionless behaviour patterns and indicators of poor welfare (Mason, 1991). In particular, locomotory stereotypy (e.g., head shaking and pacing) and oral stereotypy (e.g., bar mouthing, self-biting) have been described (Padalino et al., 2014). In the latter study, it appeared that the incidence of stereotypy decreased when the animals were exposed for one hour (h) to the females, while the frequency of typical sexual behaviors (e.g., dulaa extrusion, blathering, and teeth grinding) increased. However, whenever the bulls were exposed to female 24 h daily, they showed less stereotypical behaviors but also drastically reduced feeding and rumination, thus losing weight (Fatnassi et al., 2016). During the breeding season, bulls become very aggressive towards other males or humans and for this reason they are traditionally kept in a single box, tethered with ropes and with limited exposure to the females (Elwishy, 1988). Consequently, to date it is still a matter of debate how best to manage male camels in captivity. While the need for research to enhance camel management and safeguard their welfare has been raised (Fatnassi et al., 2016; Padalino et al., 2014), a better understanding of their daily rhythms of hormonal and behavioral patterns might be useful to adapt their management to their daily needs.

To our knowledge, no study has been performed on the daily rhythmicity of cortisol, testosterone or behavioral variables in captive camels. The aim of this study was to investigate whether male housed camels present daily rhythms of both cortisol and testosterone secretion and behavioral repertoire, including stereotypies. It was hypothesized that housed male camels might have a daily rhythmicity of cortisol, testosterone and behavioral repertoire. Knowledge of daily cortisol pattern could provide new perspectives in terms of camel welfare since this is currently regarded as a stress hormone (Bergeron et al., 2002; Haubenhofer & Kirchengast, 2007; Schmidt et al., 2010). While testosterone influences male camel libido (Deen, 2008; Monaco et al., 2015; Yagil & Etzion, 1980), a better understanding of the testosterone secretion pattern over the course of the day might help improve reproductive performance in male camels. Knowledge about the daily pattern of the behaviors, with emphasis on stereotypies, could provide information on how management systems influence camel behavior and affect their welfare. Our findings could lead to recommendations to adapt the management system of dromedary bulls for better reproductive performance (i.e., fertility rate, sperm quality), health and welfare.

## Material and Methods

The experiments were conducted according to protocols approved by the Italian Ministry for Scientific Research in accordance with EU Directive 2010/63/EU and were approved by the Animal Ethics Committee for Clinical and Husbandry Research at the Department of Emergency and Organ Transplantation (D.E.T.O.), Veterinary Clinics and Animal Production Section, DETO/168/2016.

### Animals

Six clinically healthy male dromedary camels (*Camelus dromedarius*), ranging in age from 5 to 17 years, with a mean body weight of 545 ± 63 kg and good body condition score (3.8 ± 0.7 arbitrary units; from 0 to 5 in accordance with Faye 2001), were used for this study. All animals were identified by ear tags: #808, #514, #515, #504, #373 and #3.

In summer, the bulls were kept in a single open-air paddock shaded by trees whereas, starting from the 1st of October, they were put into a traditional stable containing 6 boxes side-by-side in a line. Each box (Height = 3 m, Length = 5 m and Width = 3 m) had a roof made from insulating sheets, brick walls, and a sand floor. The bulls were tethered with a rope on the fetlock of the foreleg and were able to walk around inside the box. The doors were eastward-pointing gates, facing an open-air paddock. Each box had 6 windows, a big one on the same side as the gate and the others on the opposite wall (i.e., 2 windows in the upper section of the wall and 3 in the lower section). The gates were made out of iron bars; the camels were able to put their heads outside the box through either the bars or the big window, which enabled them to see and interact with each other (Fig. S1). The boxes were located far from the females’ pen, preventing them from seeing and touching any dams; however, at 9.00 am the female herd would pass behind the males’ boxes to go to pasture. This was the only opportunity which the males had to look at the females, either through the small windows whilst they were in their boxes or over the wall when being kept in the open-air paddock.

All year round, the camels were fed with 6 kg oat hay at 8.00 a.m., and 3 kg concentrate supplement based on barley (60%), wheat bran (17.5%), olive cake (17.5%) and a mineral and vitamin complex (5%) at 2.00 p.m. The chemical composition of the oat hay was: Dry Matter (DM) = 88.1%, Crude Protein (CP) = 6.81%, Ash = 7.9%. Dry matter content of the concentrate was 89.1% and its chemical composition was CP = 11.4%; Acid-detergent fiber (ADF) = 13.2%; Neutral-detergent fiber (NDF) = 31.6% and Ash = 8.1%. The animals were watered every day at 2.00 pm. The watering and the feeding quantity and quality remained the same throughout the experiment.

### Experimental protocol

The experiment lasted 3 days and was conducted from 15–17 December 2013 at the Arid Lands Institute’s experimental station in Médenine, Tunisia (33°30′N, 10°40′E), 18 m above sea level, thus at the onset of the breeding season. On these three days, sunrise occurred at approximately 07.17 h and sunset at 17.14 h (i.e., photoperiod of 10L: 14D).

On day 1, indwelling jugular catheters were inserted once slight sedation of the animals (5 mg/100 kg b.w. of Acepromazine, Combistress^®^, Belgium), surgical preparation of the neck, and local anesthesia (Lidocaine 2%, Unimed, Tunisia) had been conducted at the catheter insertion site. The catheters were regularly flushed with heparin solution (20 I.U./ml) throughout the experimental period.

**Figure 1:**
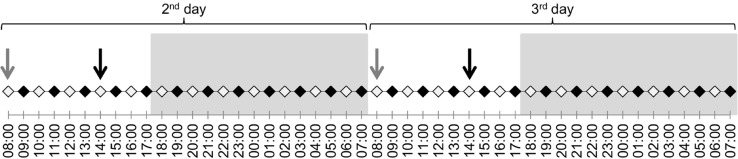
Protocol of the experiment on days 2 and 3 (16th and 17th of December). The black dots represent the blood samples and the white ones the 20-minute behavioral observations. The arrows represent the food distribution time (in grey for hay and in black for concentrate). The grey backgrounds represent the night and the white the daylight period during the experiment.

On days 2 and 3, the animals were observed for the first twenty minutes of the odd hour every two hours (i.e., from 9.00 to 9.20 am) and blood samples were collected at the beginning of even hours (i.e., at 8.00 am), with blood sample collection before food administration (Fig. 1). This decision was made to ensure that the handling required to collect the blood samples would not alter the behavior, which was filmed at the beginning of the following hour (i.e., about 55 min later).

Climatic parameters were collected by a weather station (Kestrel: 4000 Pocket Weather Tracker; Nielsen-Kellerman, USA) during the two days of behavioral observation and blood sampling (16th and 17th of December 2013) and are presented in Table 1.

**Table 1:** Climatic parameters for days 2 and 3.

	Temperature (° C)					
Day	Mean	Maximal	Minimal	Humidity (%)	Wind (m/s)	Rain (mm)
2	10.23	17.08	4.21	76.81	0.58	0
3	11.15	17.35	6.7	72.51	0.66	0

### Behavioral parameters

**Figure 2:**
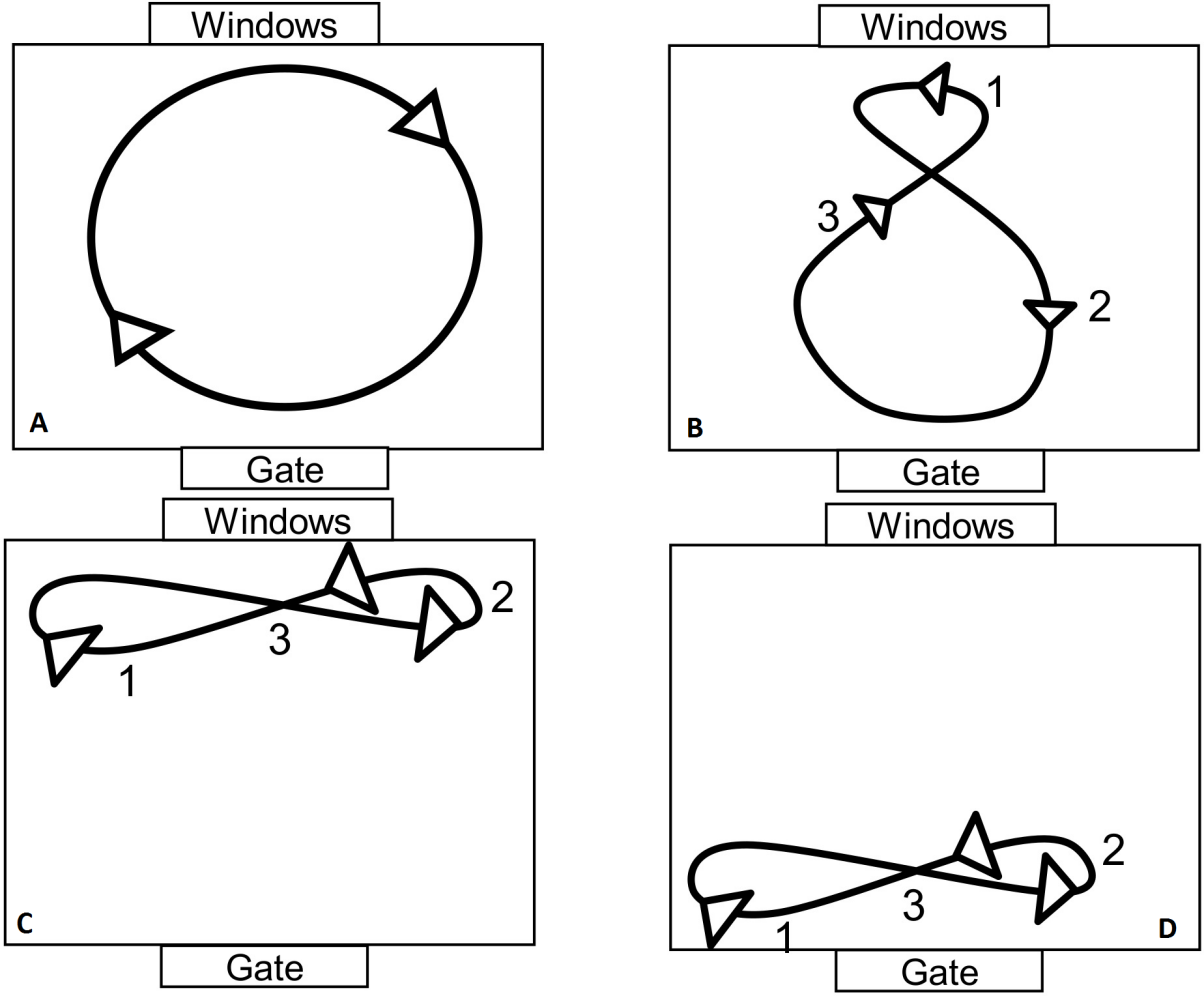
The different pathways following by the camels during box-walking stereotypy. (A) Box-walking doing a circle around the box, (B) box walking during a 8 using all the box, (C) box-walking doing a small 8 close to the window, (D) box-walking doing a small 8 close to the gate.

The videos were analyzed though a scan sampling ethogram by an expert ethologist who was blinded to the aim of the trial. The ethogram is presented in Table 2. Behaviors potentially lasting more than one minute were categorized as behavioral states and their duration was noticed (in seconds/20 min), while pinpoint behaviors were categorized as events, and their frequency was noticed (*n*∕20 min) (Altmann, 1974). Box walking (see Fig. 2 for the pathways), wall-licking, swaying, were categorized as stereotypical states while bar mouthing and head shaking were categorized as stereotypical events as had been done for such behaviors in a previous study (Padalino et al., 2014). Blathering, dulaa, teeth grinding, occipital gland scratching, flehmen and tail flapping were categorized as sexual behaviors, as in previous studies (Bhakat, Raghavendra & Sahani, 2005; Fatnassi et al., 2014; Ghoneim et al., 2012; Padalino et al., 2013; Vyas et al., 2001).

**Table 2:** Ethogram used to score the video (modified from Padalino et al. (2014) and Fatnassi et al. (2014)).

Behavior	Description
***Behavioral states***	
Feeding	Camel takes food into his mouth (hay or concentrate), chews and swallows it.
Rumination	A bolus goes back into his mouth and the camel chews it.
Walking	Camel does more than 2 complete steps.
Standing	Camel stands on his four (or three) feet and looks outside the box or apparently does nothing.
Lying down	Camel sits in sternal recumbency: sitting up on the brisket with the legs tucked under the body, the natural sitting position in camel, with head on the floor or not. Camel rests or sleeps and does nothing else.
Stereotypy	**Wall-licking**: Repetitive licking of the wall (tongue movements with contact on the wall of the box).
	**Box walking**: Walking round and round in the box in the same direction, in alternate directions or in a figure of 8. Behavior is repeated regularly and in an unvaried manner (see Fig. 2).
	**Swaying**: Camel sways his body slightly from one side to the other (i.e., Camel remains stationary but shifts his weight from one foreleg to the other and swings his head from side to side).
***Behavioral events***	
Head out	Camel puts his whole head, or part of it, through a window or through the bars on the door.
Sniffing	Exploration of the environment bringing the nose into contact with an object (e.g., the wall of the box).
Sound emission	Camel emits a sound from his mouth (sound is different from blathering).
Defecation	Elimination of feces.
Urination	Elimination of urine.
Scratching	Camel scratches a part of his body on external object (e.g., bars, wall, floor) or with another body part (e.g., scratches his head with his foot).
Yawning	Involuntary sequence consisting of mouth opening, deep inspiration, brief apnea, and slow expiration.
Open leg	Camel splays his hind legs until they form an angle of at least 45°.
Interaction with male	Camel comes into physical contact with another neighboring male (usually nose-to-nose contact, over the wall separating two boxes or through a window).
Stereotypy	**Bar mouthing**: Licking, biting or playing with the lips and/or tongue on the bars of the door.
	**Head shaking**: Camel bends his necks backwards in a very fast movement (including head movements of up to 90°). This stereotypy was considered as a behavioral event because it lasted only about one second.
Sexual behaviors	**Scratching occipital gland**: Camel scratches his occipital gland on any surface (floor, wall …) in order to spread the dark secretion of the occipital poll glands.
	**Teeth grinding**: Camel moves his lower jaw left and right, with mouth closed, grinding the teeth and producing a typical squeaking/whistling sound.
	**Blathering**: Emission of typical gurgling and roaring sounds.
	**Dulaa extrusion**: Exteriorization of the soft palate, usually known as the “dulaa”.
	**Flehmen**: Camel lifts his head and curls his upper lip.
	**Tail flapping**: The tail is held under the prepuce opening for a few seconds, and then, it is beaten up and down several times, usually spreading urine over the croup and surrounding areas.

### Blood collection and hormonal analysis

Blood samples were collected through the catheters using syringes and then drawn into Venoject^®^ tubes (Terumo Europe, Leuven, Belgium) with lithium heparin, and kept in ice until plasma was separated. Plasma samples were obtained by centrifugation at 4°C for 15 min at 3,000 rpm, within 2 h of collection, and then stored at −20°C until analyzed. During blood collection, animals were restrained gently only with halter and rope by an expert handler, who stroked the animals during the procedure. All animals thus accepted the procedure, without any signs of distress, pain or aggressiveness.

Plasma testosterone concentrations were determined in duplicate by radioimmunoassay (RIA) (Immunotech Beckman Coulter Company, Ref 1087, Marseille France). Sensitivity was 0.04 ng/ml and intra-and interassay coefficients of variation were 7.4% and 11.1%, respectively. The results were expressed in ng/ml.

Plasma cortisol level was measured by the ELISA method using a commercial cortisol ELISA kit (Demeditec Cortisol ELISA DE 1887; Demeditec Diagnostic GmbH, Kiel, Germany). All samples were analyzed in duplicate. Absorbance was measured using a Multiscan reader (basic robotic immunoassay operator, BRIO; Radim, Pomezia, Rome, Italy). The intra- and interassay coefficients of variation (CV) for plasma cortisol determined in the laboratory amounted to 5% and 7%, respectively. The results were expressed in ng/ml.

### Statistical analysis

Normal distribution was checked using the Anderson–Darling test using GenStat 64-bit Release 16.2., and, when distribution was not normal, data were log-transformed. Descriptive statistical analysis was performed using GenStat 64-bit Release 16.2.

The diurnality index was calculated for each behavioral state by dividing the mean duration of the behaviors during the photophase by the mean duration of the whole day (mean of the photophase and scotophase). The diurnal index is comprised between 0 and 1. The dashed line represents the theoretical limit between nocturnal and diurnal pattern (i.e., an index of 0.5 indicates that the behavior was performed equally as often during both the photophase and the scotophase). An index of 1 indicates that the behavior was only performed during the photophase and an index of 0 indicates that it was only observed during the scotophase (modified by Piccione et al., 2010).

Cosinor analysis was performed for the 6 behavioral states, and cortisol and testosterone levels were measured in order to investigate whether these variables present a daily rhythmicity. The analyses were performed using Refinetti, Cornélissen & Halberg’s (2007) Cosinor program (version 2.3).

All data were also assessed through a PROC mixed procedure (SAS, version 9, 1999). In the model, camel and day were used as random effects and hour was specified as a fixed factor. The Least Significant Difference (LSD) test was used to perform multiple statistical comparison (*P* < 0.05). Results are presented as least square means ± standard error (SE).

Pearson correlations were performed to identify possible associations between behavioral and hormonal parameters. The sum of all stereotypical events and all sexual behaviors was calculated and added to the model.

## Results

### Descriptive analysis

#### Behavioral states

The descriptive statistics results concerning the 6 behavioral states are presented in Table 3. Camels spent 42% of their time budget lying down and only 2% walking. Stereotypy occupied 15% of the time budget, with swaying and wall-licking being predominant. When considering the time budget during the scotophase, lying down and rumination were the two principal behaviors observed, respectively 63% and 21% of the time. The time budget of the photophase appeared to be different from the night. Indeed, lying down and rumination occupied only 14.26% and 2.08% of the time, whereas feeding, stereotypy and standing were observed respectively 30.33%, 24.25% and 25.16% of the time.

**Table 3:** Presentation of the results of the behavioral states over the two days.

Behavioral states	Time budget (%)	Mean duration (s)	SE	Time budget during scotophase (%)	Time budget during photophase (%)
Feeding	14.27	171.27	26.67	2.80	30.33
Rumination	12.92	155.03	27.36	20.66	2.08
Walking	2.03	24.39	3.84	0.68	3.92
Stereotypy	15.02	180.29	27.56	8.43	24.25
Standing	13.29	159.43	22.14	4.80	25.16
Lying down	42.47	509.59	44.71	62.62	14.26

The means represent the mean of the duration in seconds of the behavior over the 20 min of observation; SE is the standard error of the mean. The time budgets are presented as percentages (%).

The results for the different forms of stereotypy (swaying, wall-licking and box walking) are presented in Table 4. Five camels exhibited at least one of these three forms of stereotypic behaviors in this study (Table 4) and one of the camels (#3) did not perform any stereotypy.

**Table 4:** Duration (in seconds) of the different stereotypic behaviors.

Stereotypy	Mean (in s)	SE	Time budget (in %)	Min (in s)	Max (in s)	Camels
Swaying	81.05	21.81	6.75	0	1156	#515
Wall-licking	78.74	17.16	6.56	0	947	#504; #514; #515; #808
Box walking	18.18	5.24	1.52	0	385	#373; #504; #514

**Notes.**

SE is the standard error of the mean.

### Behavioral events

The descriptive statistics results concerning behavioral events are presented in Table 5.

**Table 5:** Frequency (*n*∕20 min) of the behavioral events.

	Mean	SE	Min	Max
Behavior				
Head out	2.03	0.32	0	27
Yawning	1.51	0.25	0	13
Splayed legs	0.99	0.26	0	23
Scratching	0.44	0.07	0	5
Sniffing	0.31	0.08	0	7
Defecation	0.22	0.04	0	2
Sound emission	0.17	0.07	0	8
Urination	0.14	0.03	0	2
Interaction with male	0.07	0.03	0	3
Sexual behaviors				
Teeth grinding	1.09	0.55	0	69
Blathering	0.33	0.09	0	7
Dulaa	0.24	0.07	0	6
Tail flapping	0.24	0.19	0	27
Occipital gland scratching	0.15	0.05	0	4
Flehmen	0.01	0.01	0	1

**Notes.**

SE is the standard error of the mean.

Four camels exhibited at least one of these two forms of stereotypic behavioral events (see Table 6), while camels #3 and #515 were not observed performing these stereotypic behaviors.

**Table 6:** Frequency (*n*∕20 min) of the different stereotypic behaviors.

	Mean	SE	Min	Max	Camels
Bar mouthing	0.58	0.31	0	42	#373; #504; #514; #808
Head up	0.25	0.10	0	8	#504

### Hormones

The two daily average data for testosterone and cortisol levels in each individual are presented in Table 7. The mean concentration of cortisol and testosterone over the two days was 18.55(±0.66) ng/ml and 9.01(±0.50) ng/ml, respectively.

**Table 7:** Age, mean cortisol and testosterone levels (ng/ml) for each individual.

Camel	3	373	504	514	515	808
Age (years)	17	6	8	11	11	5
Cortisol	18.46	25.09	17.26	15.46	16.17	18.89
Testosterone	3.29	5.56	7.31	13.47	23.06	1.35

### Diurnality index

The indices for rumination and lying down are close to 0 (respectively, 0.09 and 0.19), meaning that these two behaviors were mainly performed at night. The indices for stereotypy, standing, feeding and walking are close to 1 (respectively, 0.74, 0.84, 0.92 and 0.85), meaning that these behaviors were mainly performed during the photophase of the dark-light cycle (Fig. S2).

### Cosinor results

All behavioral states show a 24 h-period rhythmicity (*P* < 0.05), with resting behavior presenting the strongest robustness (41%) and stereotypy the weakest (9.2%). Concerning hormonal data, cortisol levels show 24 h periodicity with 7.7% robustness, whereas testosterone levels showed no rhythmicity (*P* = 0.95). The acrophase of lying down and rumination occurred at night (respectively, 23:07 and 02:54) whereas the acrophase of feeding, standing, walking, stereotypy and cortisol occurred during daylight around midday (more or less one hour) (Table S1).

### Effect of hour

#### Behavioral states

**Figure 3:**
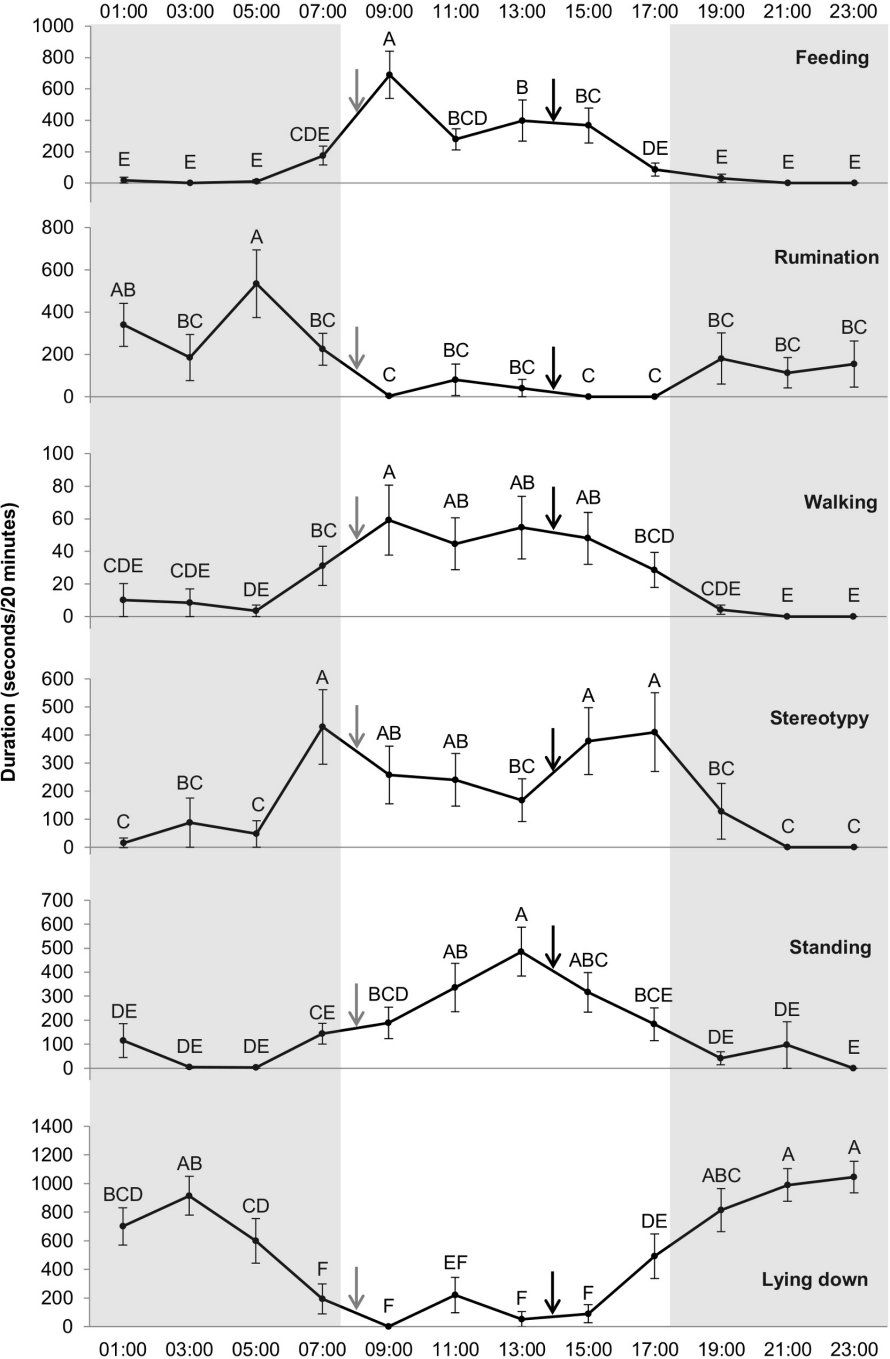
Duration of the different behavioral states (in seconds/20 min) for each hour. Grey and white backgrounds represent the dark and light periods of the day, respectively. Arrows represent food distribution times (grey for hay and black for concentrate). Values within the same graph with the same superscript letter are not significantly different from one another (*P* > 0.05).

The mixed procedure showed a significant effect of the hour on expression of the 6 behavioral states: lying down (*df* = 11, *F* = 14.08, *p* < 0.0001), feeding (*df* = 11, *F* = 10.24, *p* < 0.0001), stereotypy (*df* = 11, *F* = 5.53, *p* < 0.0001), walking (*df* = 11, *F* = 5.46, *p* < 0.0001), standing (*df* = 11, *F* = 5.25, *p* < 0.0001), rumination (*df* = 11, *F* = 3.40, *p* < 0.001). Figure 3 shows the time spent by the camels in the different behavioral states for each hour and the differences between the hours. Rumination was higher at 5:00 (534.42 ± 160.2 s) compared to all the other hours (*p* < 0.05), except 1:00 (339.92 ± 101.6 s). Feeding was higher at 9:00 than the rest of the day. Feeding and walking duration were both higher at 9:00 (after hay was distributed at 8:00) compared to 7:00 (689.67 ± 150 *vs* 175.54 ± 59.7 and 59.17 ± 21.5 *vs* 31.18 ± 12 s, *p* < 0.0001 and *p* = 0.0434, respectively). Feeding and walking were not observed during several hours of the night. Stereotypies were performed more frequently at 15:00 compared to 13:00 (378.59 ± 116.9 *vs* 167.83 ± 67.2 s, *p* = 0.0317). Stereotypic behavior duration increased after the distribution of the concentrate at 14:00. Stereotypies were performed more often at 7:00 compared to 5:00 (428.85 ± 130.6 *vs* 47.67 ± 47.7 s, *p* = 0.0001); stereotypies increased in frequency just before food distribution at 8:00. Moreover, camels did not perform any stereotypic behaviors during the observations at 21:00 and 23:00, whereas stereotypies were observed throughout the light period (with at least a duration of 167.83 s/20 min).

**Figure 4:**
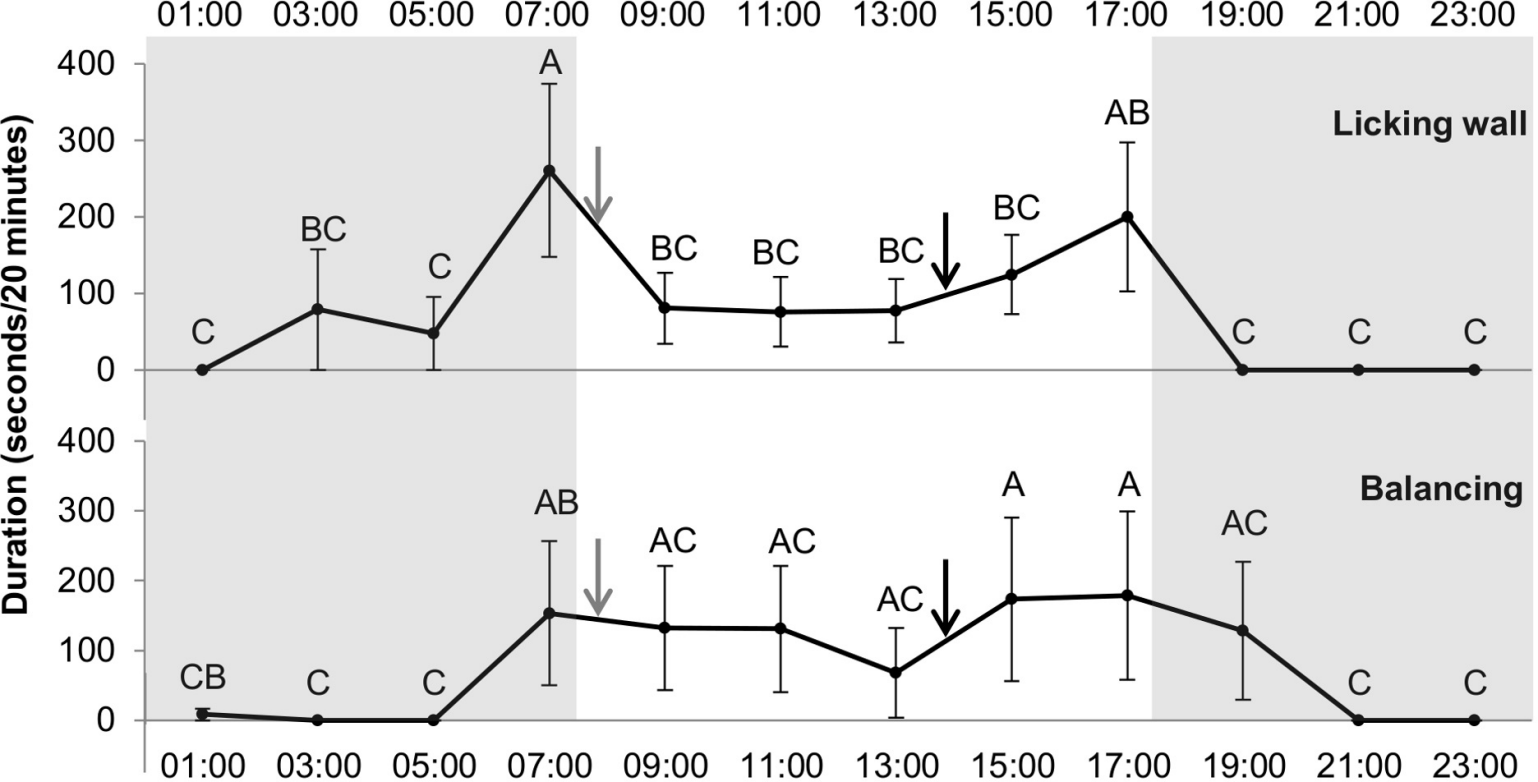
Duration of licking wall and balancing (stereotypy) (in seconds/20 min) for each hour. Grey and white backgrounds represent the dark and the light period of the day, respectively. Arrows represent food distribution times (grey for hay and black for concentrate). Values within the same graph with the same superscript letter are not significantly different from one another (*P* > 0.05).

Concerning stereotypies, an effect of the hour was found for swaying and wall-licking (*df* = 11, *F* = 1.97, *p* < 0.05; *df* = 11, *F* = 3.08, *p* < 0.0001, respectively) whereas hour had no significant effect on box walking. Figure 4 presents the time spent swaying and wall-licking for each hour and the differences between the hours. The two stereotypies were higher at 7:00 compared to 5:00 (i.e., the duration increased just before food distribution at 8:00). Moreover, wall-licking duration was higher at 7:00 than 9:00 (260.76 ± 113.3 *vs* 80.58 ± 46.5 s, *p* = 0.008) and decreased after hay distribution at 8:00. These two different forms of stereotypies (swaying and wall-licking) were not observed during some hours of the night but were always observed during daylight hours.

#### Behavioral events

The mixed procedure showed a significant effect of hour on expression for the following behaviors: head out (*df* = 11, *F* = 4.92, *p* < 0.0001), defecation (*df* = 11, *F* = 5.88, *p* < 0.0001), urination (*df* = 11, *F* = 2.69, *p* < 0.01), yawning (*df* = 11, *F* = 2.8, *p* < 0.01), blathering (*df* = 11, *F* = 4.3, *p* < 0.0001), dulaa (*df* = 11, *F* = 4.38, *p* < 0.0001), splayed legs (*df* = 11, *F* = 2.23, *p* < 0.05) and interaction with male (*df* = 11, *F* = 2.55, *p* < 0.01). No effect of hour on expression of the following behaviors was found (*p* > 0.05): sniffing, sound emission, scratching, occipital gland scratching, teeth grinding, flehmen and tail flapping.

**Figure 5:**
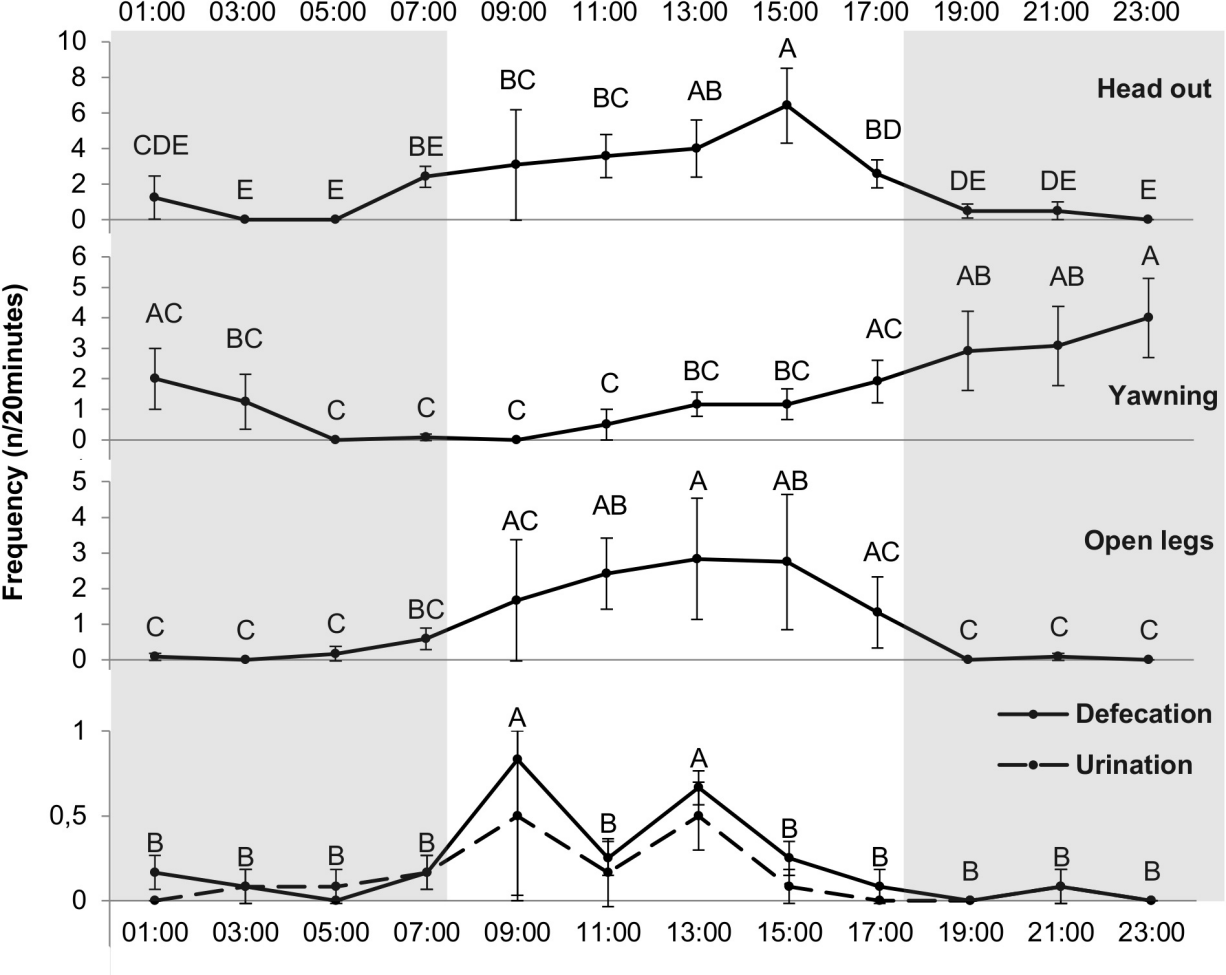
Frequency of different behavioral events (*n*∕20 min) for each hour. Grey and white backgrounds represent the dark and the light period of the day, respectively. Arrows represent food distribution times (grey for hay and black for concentrate). Values within the same graph with the same superscript letter are not significantly different from one another (*p* > 0.05).

For the “interaction with male” behavior, only one hour differed from the others (frequency at 7:00 was higher than all other hours, *p* < 0.05). Concerning sexual behaviors, blathering and dulaa frequencies presented the same pattern, peaking at 9:00. Figure 5 presents the frequency for each hour of the day, and the differences between the hours, of the following behavioral events: head out, yawning, splayed legs, defecation and urination. Urination and defecation are combined in Fig. 5 as they presented the same pattern: 9:00 and 13:00 were higher than other hours. The camels yawned more at the beginning of night (19:00, 21:00 and 23:00) that at the end of night and beginning of day (5:00, 7:00, 9:00, 11:00). Splayed legs frequency was higher around midday (11:00, 13:00 and 15:00) than at night (19:00, 21:00, 23:00, 1:00, 3:00 and 5:00). No effect of hour was found for head up and bar mouthing frequency (*p* > 0.05).

#### Hormones

**Figure 6:**
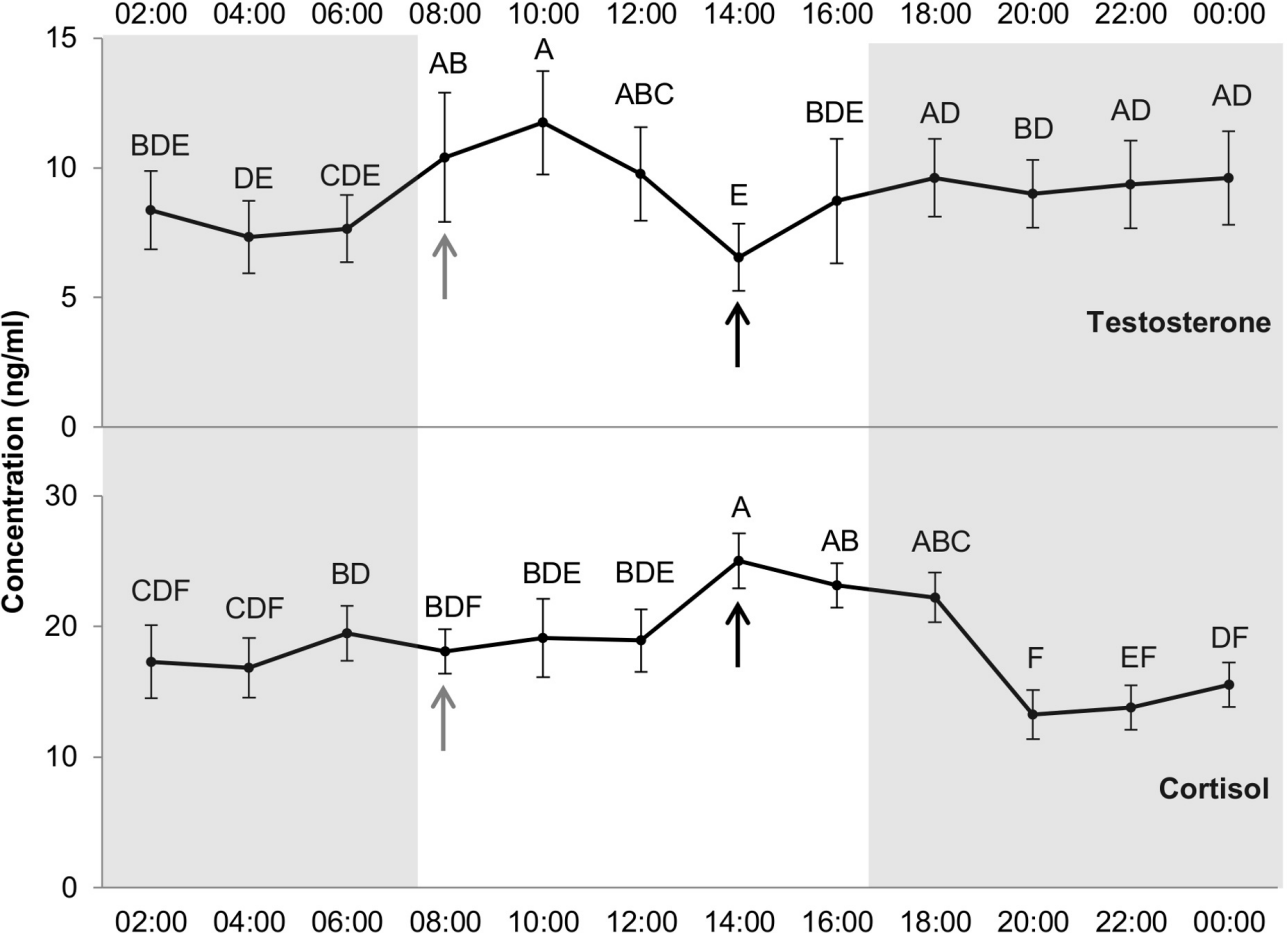
Levels of testosterone and cortisol (ng/ml) per hour. Grey and white backgrounds represent the dark and the light period of the day, respectively. Arrows represent food distribution times (grey for hay and black for concentrate). Values within the same graph with the same superscript letter are not significantly different from one another (*p* > 0.05).

Effect of hour was significant on cortisol and testosterone levels (*df* = 11, *F* = 3.3, *p* < 0.001; *df* = 11, *F* = 2.69, *p* < 0.01, respectively). Differences between the hours of the day were also found for both hormones (Fig. 6). Cortisol level peaked at 14:00 (25.02 ± 2.1 ng/ml) remaining steady at 16:00 and 18:00 and then falling. The minimum cortisol value was observed at 20:00 (13.25 ± 1.9 ng/ml), with an increase between 12:00 and 14:00, just before concentrate distribution (just after blood sampling at 14:00). The maximal value for testosterone was observed at 10:00 (11.74 ± 2.0 ng/ml) and the minimal at 14:00 (6.55 ± 1.3 ng/ml).

### Pearson correlations

Cortisol was negatively correlated with stereotypy duration (*r* =  − 0.287; *P* = 0.005) and frequency (*r* =  − 0.171; *P* = 0.040).

Testosterone was positively correlated with the frequency of the sum of all sexual behaviors (*r* = 0.164; *P* = 0.040), blathering and dulaa frequency (*r* = 0.198; *P* = 0.017; *r* = 0.171; *P* = 0.039, respectively). Testosterone was also negatively correlated with cortisol (*r* =  − 0.297; *P* = 0.003).

Other correlations were not significant.

## Discussion

This study reports on the daily rhythms of cortisol and testosterone secretion and behavioral repertoire in housed male dromedary camels. Our results support the hypothesis that camels have a daily rhythmicity of cortisol secretion and expression of behaviors. Daily rhythmicity for testosterone was not found, but these results need to be ascertained using a large dataset. Our results suggest that cortisol secretion and behavior manifestation were influenced not only by light-dark cycle but also by food distribution, which also acts as a Zeitgeber in the housed camel bulls. While stereotypies were mostly performed around the hour of food delivery, scheduled restrictive feeding appeared to be a source of frustration for these animals and failed to respect the welfare principle of good feeding. However, this hypothesis needs to be further investigated comparing the effects of restrictive and *ad libitum* feeding on the daily rhythms of camel bulls.

The Cosinor analysis revealed a 24 h periodicity for the six behavioral states with different acrophase timing (Table S1). The acrophase of lying down and rumination (considered as inactivity as for Bushbuck (Wronski, Apio & Plath, 2006)) occurred at night whereas the acrophase of activities (feeding, stereotypy and walking) occurred during the light phase of the day and, more precisely, around midday. The rhythmicity of rumination is in accordance with the literature; Gordon & McAllister (1970) found a 24 h-period rhythmicity for rumination in sheep, with higher values observed in the second half of a 12 h dark period. In camels, it has been reported that rumination starts after midnight and lasts until 8:00, reaching peak values between 1:00 and 6:00 (Kaske et al., 1989) or between 4:00 and 7:00 (Iqbal & Khan, 2001). Our findings on daily activity patterns are also in agreement with previous studies. Locomotor activities of horses, goats, sheep and cows, housed individually in boxes, showed acrophase at the middle of the photoperiod (Bertolucci et al., 2008; Piccione, Caola & Refinetti, 2005; Piccione et al., 2008a; Piccione et al., 2008b; Piccione et al., 2010). An increase in relative activity from sunrise, reaching a peak toward midday, and then gradually decreasing in activity until sunset has also been described in wild Bactrian camels (Xue et al., 2015). With regard to the diurnality index, all activities (feeding, stereotypy and walking) presented a diurnal pattern. Thus, the camels appeared to be more active during the photophase.

Our findings revealed that camels were more active during daylight and comparatively inactive at night; lying down and rumination accounted for 62% and 21% of the time budget at night, respectively. As a management system should meet their behavioral needs, camels should be provided with a quiet night-time environment to rest and ruminate and be kept on pasture or in the paddock during the day. Our management system seems inadequate during the day. The camels were more active during the day with a peak of activity around midday, but in their box they could not walk and forage enough. During the day camels at pasture have been shown to spend 24% of the time walking and 61% feeding with a total of 30 km covered per day (Chaibou, 2005), whereas in our study, during the photophase, walking made up only around 4% and feeding 30% of the time budget. Camels allowed to forage in the savanna spent 21.2% of the time walking and 71.1% feeding (O’Connor, 2013). Similarly, camels allowed to graze 8 h per day spent 37.41% grazing, 31.7% rumination, 26.52% idling and 4.32% resting over a 24-hour period (Khan et al., 1998). In our study, the camels spent less time feeding, walking and ruminating over the whole day compared to these studies and more time lying down. These differences are related to management, because the restricted space and access to food did not allow the camels to have the same time budget as individuals at pasture. Our camels were fed with limited amounts of hay and with concentrates which were rapidly ingested. It is known that under high-fibre alimentation with low digestibility, camels need more rumination time in order to digest this diet (Von Engelhardt et al., 2006). So, feeding practices could explain the limited time spent feeding and ruminating by the present camels, thus explaining the differences with previous studies. Indeed, in camels kept in barns, fed with hay-based diets (with hay *ad libitum*), feeding took up 23.3% of the day and rumination 34.6%. Likewise, Bactrian camels fed with hay *ad libitum* ruminated 22% of their time (Cahill & McBride, 1995) and camels housed in open barns with straw *ad libitum* ruminated 40% and fed 30% (Hedi & Khemais, 1990). Therefore, providing more hay or a higher fibre-diet, slower to ingest and digest than concentrate, could help increase feeding and rumination times in camels and probably decrease feeding frustration. Providing straw reduced feeding motivation and increased explorative, foraging and feeding behaviors in pigs; feeding and rumination in cattle (Tuyttens, 2005). Providing straw bedding should be recommended for camels housed in boxes, in order to fulfill their behavioral needs to forage and feed. Moreover, providing camels with access to a paddock (ideally pasture), during daylight (more particularly around midday), where they can graze and walk seems necessary to allow them to express their natural behavioral patterns.

The behavioral rhythmicity observed in this study may have occurred in response to the prevailing light-dark cycle or by the scheduled feeding regime as already reported in other species (Boulos & Terman, 1980; Piccione & Caola, 2002). During the experiment, the camels were fed twice daily at scheduled times with restricted rations, a system which seemed to influence in particular the daily rhythmicity of stereotypy. In our study, the camels spent 15% of the time performing stereotypic behavior. In a previous study, stereotypic behaviors amounted to around 10% of the time budget in housed male camels (Padalino et al., 2014); whereas stereotypy is not (or rarely) performed by wild animals (Mason, 1991). In some species, stereotypies are mostly performed just before meals arrive whereas in others it occurs just after food consumption (Mason & Mendl, 1997). Our results show that stereotypies are mostly performed during daylight, specifically around the hours of food distribution. Total stereotypy duration increased after concentrate distribution at 14:00. Thus, food consumption seems to lead to an increase in stereotypic behaviors in camels as has already been observed in other species (Bergeron et al., 2006; Mason & Mendl, 1997). For example, Jensen (1988) observed that pregnant sows performed most stereotypic behavior 2 h after feeding. Terlouw et al. (1993) suggest that food ingestion could stimulate stereotypic behaviors in sows. Food restriction and the impossibility to forage are known to be the main causes of oral stereotypies in pigs (Tuyttens, 2005). Lying down in pigs after feeding is considered as a sign of satiety (Bergeron et al., 2000). In our study, the camels did not lie down more frequently after food distribution while a rise in stereotypy was observed, which could be a sign that camels did not feel satiety and were frustrated after feeding. When considering the different forms of stereotypies in detail, it becomes clear that not all stereotypies presented the same pattern. Wall-licking duration increased before feeding and decreased after food distribution. Wall-licking thus occurred during pre-feeding, as has been observed in minks, in which most stereotypies were observed before food distribution (Mason, 1993). This phenomenon has also been observed in horses (Cooper & McGreevy, 2007) and is probably due to their anticipation of food arrival. Nevertheless, the window of behavioral observations was narrow (20 min every two hours, and was performed one hour before and after food distribution) and there was not comparison with camels fed ad libitum. It could be interesting to follow the behaviors of the camels continuously at food distribution times (e.g., 2 h before and after food delivery) in order to investigate more precisely the pattern of pre- and post-feeding stereotypic behaviors, comparing different feeding management. This could provide a better understanding of the kind of stereotypies that are affected by food arrival and food consumption. Overall, our housed camels exhibited five forms of stereotypic behaviors (two oral and three locomotor stereotypies) which did not follow the same daily pattern. Considering the high incidence of stereotypy, an indicator of altered welfare (Mason & Latham, 2004), and their association with feeding time, the management system in question should be considered as sub-optimal.

Interestingly, stereotypy was negatively correlated with cortisol. This finding might be interpreted taking into account that stereotypes have been considered as a coping mechanism during stressful situations, reducing cortisol secretion (Mason, 1991). The mean cortisol level over the day (18.55 ng/ml) in our camels was indeed within the normal range of cortisol plasma concentration observed in previous studies in camels during the breeding season (Ali et al., 2013; Bhakat, Raghavendra & Sahani, 2005; Fatnassi et al., 2014; Sid-Ahmed et al., 2013). Our result is also similar to the value (18.8 ± 2.0 ng/ml) obtained by Fatnassi et al. (2014) in male camels housed in the same conditions (housed in a single box 24 h per day).

Saeb et al. (2010) suggested that daily variations might affect cortisol levels in camels, since the concentration differed between the three samples made at 08:30, 09:30 and 13:30. However, no study of daily rhythm of cortisol in camels has been conducted previously, making this the first to report it. In our study, cortisol levels showed a 24 h period rhythmicity, with an acrophase at 12:57 and the lowest value at 20:00. Daily rhythmicity of cortisol secretion has been reported in many species. Thun et al. (1981) found daily rhythmicity of cortisol secretion in bulls, with the highest value measured at the onset of the light period and the lowest at the beginning of the dark period. Bohák et al. (2013) also revealed daily rhythmicity for cortisol in horses with an acrophase at 10:50 and a minimum at 22:00. Hemmann et al. (2012) showed daily rhythmicity in horses with the lowest cortisol values around 22:00–24:00 h and the highest at 08:00–14:00. Similarly, Hoffis et al. (1970) found peak cortisol values in the morning and the lowest values in the evening in horses. Irvine & Alexander (1994) observed a daily rhythm of cortisol in horses with a peak at 06:00–09:00, and a nadir at 18:00–21:00. In pigs, higher cortisol was found at 10:00 compared to 18:00 (Janssens, Helmond & Weigant, 1995). Our results therefore appear to agree with findings whereby cortisol levels are low in the evening and high in the morning (or around midday) as in other ungulates and that they are influenced by the light-dark cycle. Cortisol level is usually used in studies dealing with animal welfare as stress hormone (e.g., dogs: Bergeron et al., 2002; Haubenhofer & Kirchengast, 2007; horses: Schmidt et al., 2010; Waran, Leadon & Friend, 2007; camels: El Khasmi et al., 2015; El Khasmi et al., 2013). Our findings suggest that it seems necessary to take the samples at the same time of day within an experiment aiming to compare two cortisol levels in male dromedary camels.

It has been reported that scheduled feeding could also entrain cortisol rhythmicity. For example, in ewes fed once daily, daily rhythmicity for cortisol was observed, whereas such rhythmicity was not present in ewes fed every two hours in the day (Simonetta, Walker & McMillen, 1991). In our study, the camels were fed twice daily at precise hours of the day, so it is possible that the scheduled feeding entrained the daily rhythm of cortisol. Simonetta, Walker & McMillen (1991) noticed that the level of cortisol in ewes increased just before food distribution. We observed this phenomenon in our study, with cortisol concentration increasing from 18.92 ng/ml, at 12:00, to 25.02 ng/ml at 14:00 (taking the blood sample at 14:00 just before concentrate distribution). The link between daily rhythmicity and scheduled food distribution and ingestion remain to be investigated in camels for a better understanding of the relationship between feeding and cortisol secretion.

Cortisol was negatively correlated with testosterone. This finding is in line with the literature which suggests that poor welfare may lead to poor reproductive performance, namely lack of libido and hypo-fertility (Padalino, Monaco & Lacalandra, 2015). However, the daily mean plasma testosterone level was 9.01 ± 0.5 ng/ml, within the normal range reported in previous studies in camels during the breeding season (Al-Qarawi et al., 2000; Al-Qarawi et al., 2001; Deen, Vyas & Sahani, 2005; El-Harairy & Attia, 2010; Monaco et al., 2015). Plasma testosterone concentration may range from 2.6 ± 0.3 ng/ml (Al-Qarawi et al., 2000) to 42.14 ± 2.78 ng/ml (Deen, Vyas & Sahani, 2005), because it can be influenced by different parameters, such as diet (Mogawer et al., 2013), exposure to females (Bhakat, Raghavendra & Sahani, 2005) and age (Hassan, 2007). Al-Qarawi et al. (2000) found that young (<3 years) and old camels (>15 years) presented the lowest concentration (respectively, 1.4 ng/ml and 2.6 ± 0.3 ng/ml) and the mature (5–15 years) the highest values (4.8 ± 0.6 ng/ml). In our study, the youngest camel (5 years) and the oldest camel (17 years) both presented the lowest values (respectively, 1.35 and 3.29 ng/ml) while the highest values were observed in the mature ones. In the present study, a daily rhythm with a 24 h periodicity was not found for testosterone secretion in camels. This result is in accordance with studies in other species, such as male white-tailed deer (Bubenik et al., 1983), dogs (Thun, Eggenberger & Zerobin, 1990), rams (Falvo et al., 1975) and rabbits (Moor & Younglai, 1975; Rowe et al., 1975). However, the lack of daily secretion for testosterone might be due to the low numbers of subjects and their wide age range. Our results showed several differences in testosterone level over the day with the maximal value measured at 10:00 (11.74 ± 2.0 ng/ml) and the minimal at 14:00 (6.55 ± 1.3 ng/ml). It is noteworthy that the maximal testosterone value was measured after the passage of the female herd behind the males’ box at 9:00. It seems that visual and olfactory contact with the females had an immediate impact on their testosterone levels, stimulating their sexual behaviors. Indeed, the frequency of sexual behaviors (blathering and dulaa) peaked at 9:00 when the females passed behind the males’ box. Blathering and dulaa extrusion are considered signs of high libido in camels (Padalino et al., 2013) and, consequently, it is not surprising that they were positively correlated with testosterone in our study. Bhakat, Raghavendra & Sahani (2005) observed a higher testosterone level, over several weeks, in male camels exposed daily to females (visual contact for 30 min per day) compared to unexposed males, suggesting that exposure to females has long-term effects exposure. Our results, however, suggest that this rapid exposure to females had no impact on overall testosterone levels throughout the day because the value obtained in our study (9.01 ± 0.50 ng/ml) is quite similar to that obtained by Bhakat, Raghavendra & Sahani (2005) in male camels unexposed to females (8.42 ± 2.83 ng/ml). Overall, few minutes of female exposure would appear to be insufficient to entrain a global rise in testosterone secretion over a day in camels but, apparently, only cause a short-term increase. Nevertheless, these results suggest that the procedure described by Fatnassi et al. (2014), consisting in presenting a female to the male just before semen collection, could be a good way of increasing testosterone secretion and sexual behaviors in camels.

Our findings needs to be interpreted with caution and should be considered preliminary, because this study was limited by several factors. Firstly, only six animals of different ages were used, they were observed only for short period (2 days) and the experiment was not repeated. Individually could therefore have affected the results, especially of testosterone. Secondly, as it was not possible to compare different management systems and only one management system was tested, the data are strictly limited to that management system. Finally, as only male dromedary camels were used, our findings should be considered valid only for this species and gender. Notwithstanding these limitations, this study increased our understanding of daily rhythmicity in dromedary bulls in a captive environment. The findings could prove useful to enhance their management and welfare.

## Conclusions

This is the first study documenting that male camels housed in a single box present daily rhythmicity of behaviors and cortisol secretion but not of testosterone. The light-dark cycle and scheduled food distribution seemed to act as timing-cues. Even though the housing system did not impair daily rhythmicity, the high incidence of stereotypic behaviors indicates that housing male camels for 24 h a day should not be recommended. Giving access to a paddock during the day, frequent feeding with a fiber-rich diet, and housing overnight with straw bedding might be proposed to meet the physiological and behavioral needs of male camels and to enhance their reproductive performance, health and welfare. Further studies comparing the effects of different housing and feeding systems using hormonal and behavioral variable daily rhythmicity as welfare indicators seem necessary to find the best management system for dromedary bulls.

##  Supplemental Information

10.7717/peerj.3074/supp-1 Figure S1 Camel stable Boxes where the camels were kept.

10.7717/peerj.3074/supp-2 Figure S2 Diurnality indices for the six behavioral states Each bar indicates the diurnality index for each behavior. The dashed line indicates the theoretical separation between nocturnal and diurnal pattern.

10.7717/peerj.3074/supp-3 Table S1 Results of the Cosinor analysis

10.7717/peerj.3074/supp-4 Data S1 Raw data

## Additional Information and Declarations

### Data Availability

The following information was supplied regarding data availability: The raw data has been supplied as a Supplementary File.
