# Supplementary figures and images for: Daily rhythms of behavioral and hormonal patterns in male dromedary camels housed in boxes
Source: PeerJ. 2017 Mar 29;5:e3074. doi: 10.7717/peerj.3074 (PMC5374969; doi:10.7717/peerj.3074)

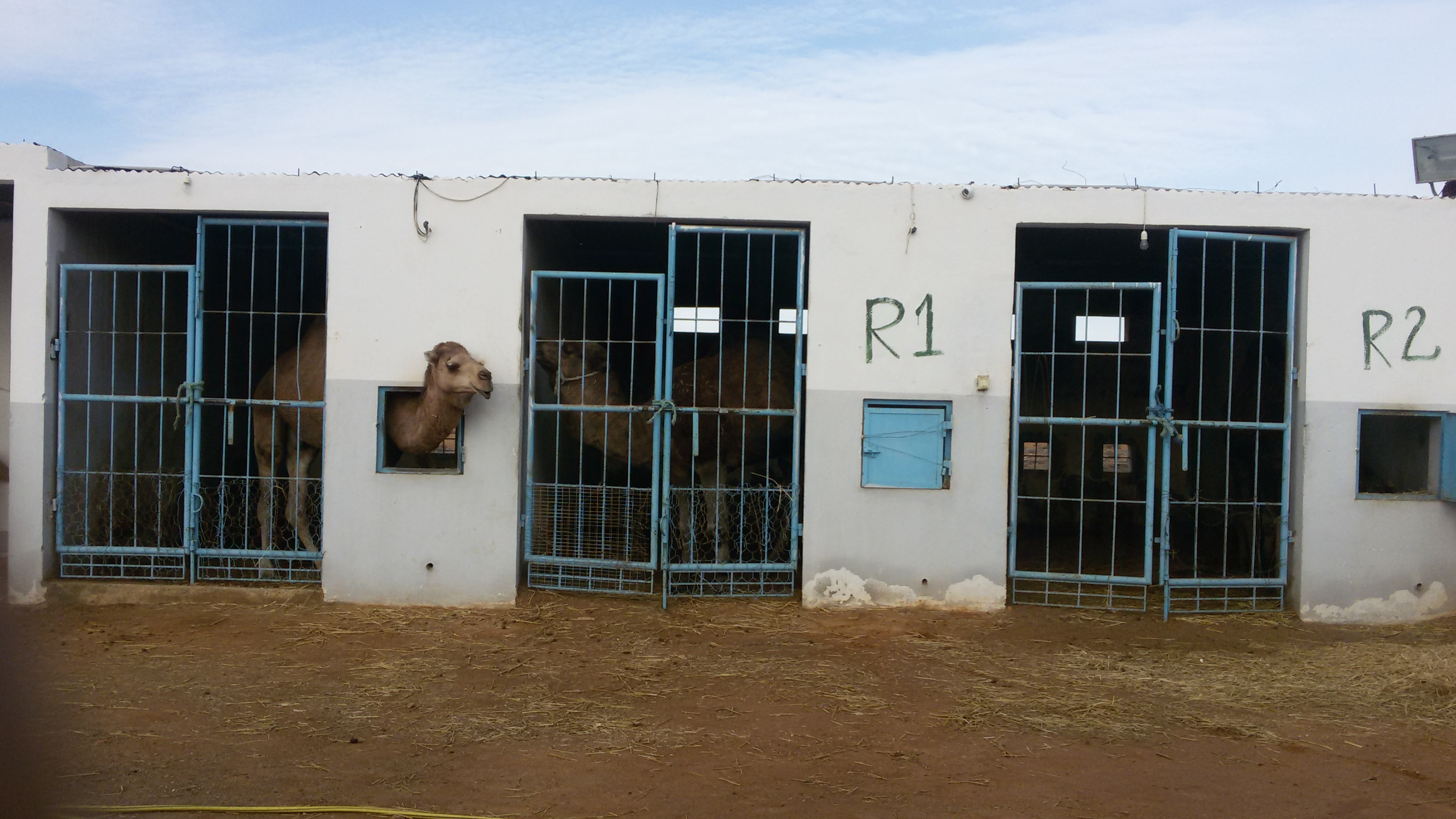

Supplement: Figure S1 — Boxes where the camels were kept. [file peerj-05-3074-s001.png]

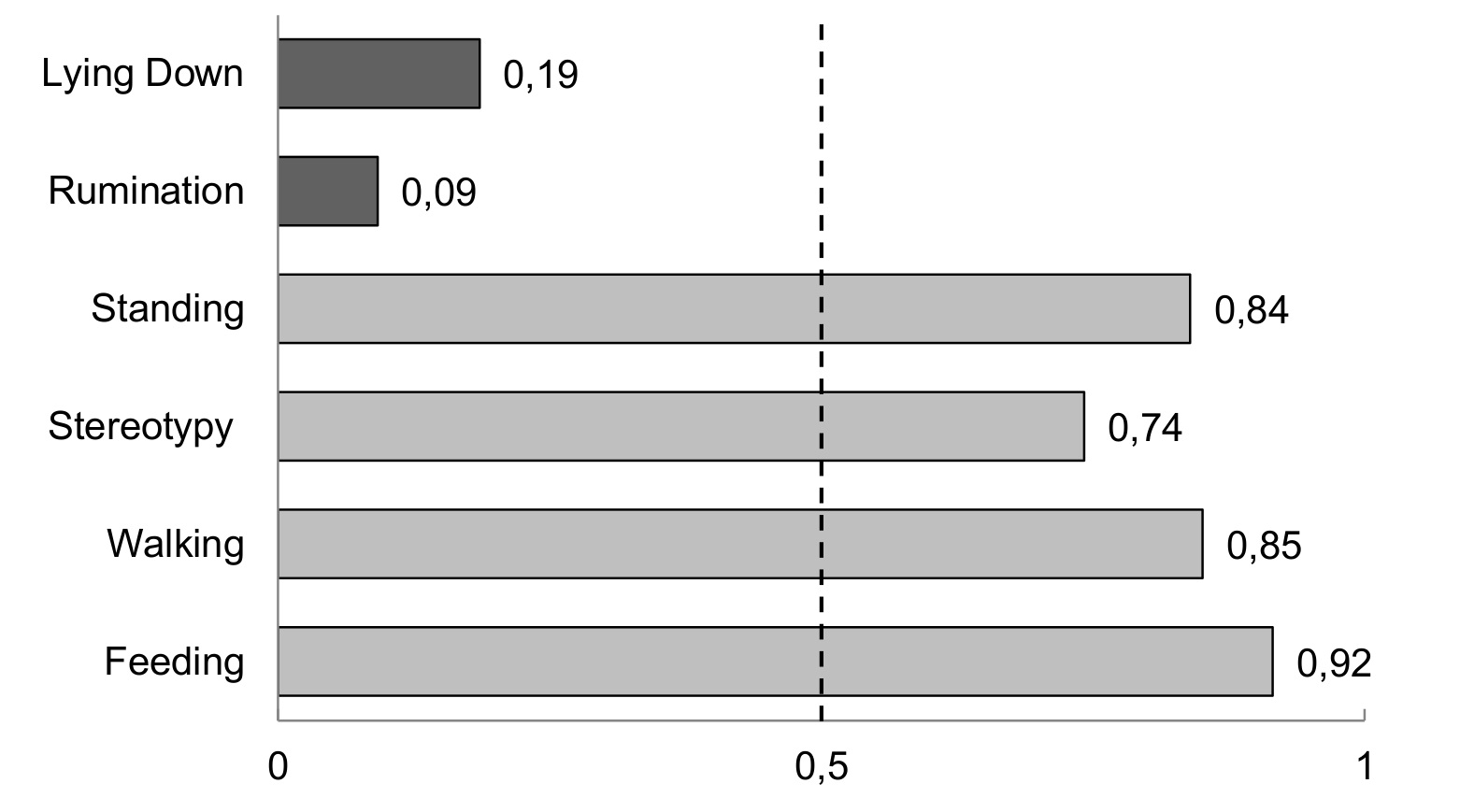

Supplement: Figure S2 — Each bar indicates the diurnality index for each behavior. The dashed line indicates the theoretical separation between nocturnal and diurnal pattern. [file peerj-05-3074-s002.png]
