# Supplementary material for: Daily rhythms of behavioral and hormonal patterns in male dromedary camels housed in boxes
Source: PeerJ. 2017 Mar 29;5:e3074. doi: 10.7717/peerj.3074 (PMC5374969; doi:10.7717/peerj.3074)
Supplement: Table S1 [file peerj-05-3074-s003.docx]

**Supplementary Table 1:** Results of the Cosinor analysis

| Behavior | Period | F(2.141) | P | Mesor | Amplitude | Acrophase | Robustness |
| --- | --- | --- | --- | --- | --- | --- | --- |
| Lying down | 24h | 51.64 | <0.0001 | 509.43 | 493.30 | 23:07 | 41.00% |
| Feeding | 24h | 28.89 | <0.0001 | 170.97 | 243.91 | 11:17 | 27.60% |
| Standing | 24h | 18.74 | <0.0001 | 158.84 | 171.55 | 12:44 | 19.30% |
| Walking | 24h | 16.42 | <0.0001 | 24.33 | 28.26 | 11:38 | 17.20% |
| Rumination | 24h | 9.10 | <0.001 | 155.03 | 157.02 | 02:54 | 9.60% |
| Stereotypy | 24h | 8.82 | <0.001 | 179.58 | 155.41 | 12:14 | 9.20% |
| Hormone |  |  |  |  |  |  |  |
| Cortisol | 24h | 7.49 | <0.01 | 18.55 | 3.49 | 12:57 | 7.70% |
| Testosterone | 24h | 0.05 | 0.95 | - | - | - | 0% |
